# An evaluation of the effect of pulse-shape on grey and white matter stimulation in the rat brain

**DOI:** 10.1038/s41598-017-19023-0

**Published:** 2018-01-15

**Authors:** Marjolijn Deprez, Kelly Luyck, Laura Luyten, Tim Tambuyzer, Bart Nuttin, Myles Mc Laughlin

**Affiliations:** 1 Experimental Neurosurgery and Neuroanatomy, Department of Neurosciences, KU Leuven, B-3000 Leuven, Belgium; 2 Centre for Psychology of Learning and Experimental Psychopathology, Department of Psychology, KU Leuven, B-3000 Leuven, Belgium; 3 Division Animal and Human Health Engineering, Department of Biosystems, KU Leuven, B-3000 Leuven, Belgium; 4 ExpORL, Department of Neurosciences, KU Leuven, B-3000 Leuven, Belgium

## Abstract

Despite the current success of neuromodulation, standard biphasic, rectangular pulse shapes may not be optimal to achieve symptom alleviation. Here, we compared stimulation efficiency (in terms of charge) between complex and standard pulses in two areas of the rat brain. In motor cortex, Gaussian and interphase gap stimulation (IPG) increased stimulation efficiency in terms of charge per phase compared with a standard pulse. Moreover, IPG stimulation of the deep mesencephalic reticular formation in freely moving rats was more efficient compared to a standard pulse. We therefore conclude that complex pulses are superior to standard stimulation, as less charge is required to achieve the same behavioral effects in a motor paradigm. These results have important implications for the understanding of electrical stimulation of the nervous system and open new perspectives for the design of the next generation of safe and efficient neural implants.

## Introduction

Electrical stimulation of the nervous system has a wide range of clinical applications, from sensory restoration delivered via retinal implants^1^, to the treatment of neurological and psychiatric disorders with deep brain stimulation^2,3^. While the clinical applications are diverse, the basic stimulation method is the same – a charge-balanced, rectangular, electric pulse. For effective neural stimulation, at the most basic level, the pulse must inject enough charge to move the membrane from resting to threshold potential, thereby causing the neuron to fire an action potential. This very basic stimulation mechanism can then lead to a cascade of more complex, often system-wide, effects^4^. On the other hand, if excessive charge is injected, tissue damage can occur^5,6^. Therefore, optimal neural stimulation seeks to reduce pulse parameters such as amplitude and charge per phase, while preserving stimulation effectiveness. Reducing the amount of charge needed to achieve the same effect, has the potential to reduce tissue damage, increase battery life or expand the therapeutic window.

**Figure 1:**
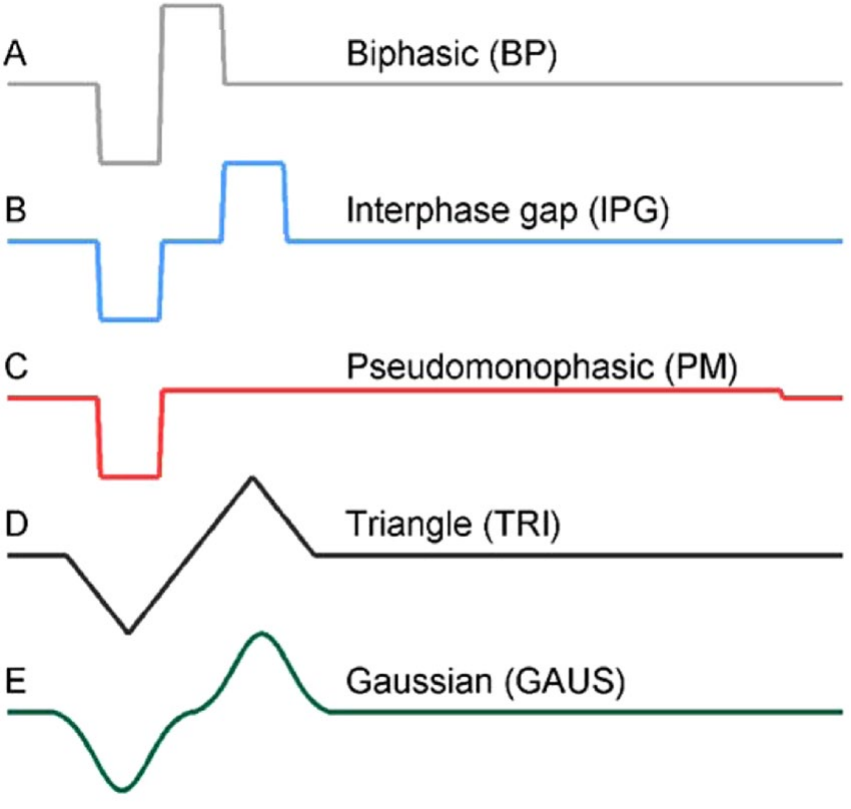
Graphical overview of the pulse-shapes studied in this paper. All pulse-shapes were designed to be charge balanced, *i.e*. the first and second phases have equal, but opposite, charge. Additionally, all pulses were designed so that for any given first phase amplitude, each pulse-shape delivers the same amount of charge per phase.

Animal and human studies of auditory nerve stimulation have shown that pulse-shape can be manipulated to improve effectiveness. For instance, introducing an interphase gap (IPG, compare Fig. 1A and B) produces an increase in stimulus audibility when compared to a biphasic pulse (BP) with equal amplitude and charge per phase^7–9^. Pseudomonophasic pulses (PM), in which the first phase is followed by a longer and lower-amplitude charge-balancing phase **(**Fig. 1C**)**, are more effective than biphasic pulses^10–12^. These findings can be partially attributed to the counteracting effects of the two opposite phases in a typical biphasic pulse: the first phase depolarizes the membrane and initiates an action potential, whereas the second phase counters this effect by rapidly hyperpolarizing the membrane. By delaying current injection in the second phase, the membrane is allowed to fully integrate the current delivered in the first phase and reach threshold.

In addition to IPG and PM stimulation, a few studies have shown that neurons can be stimulated using non-rectangular pulse-shapes. Modeling studies show that triangular **(**Fig. 1D**)**, Gaussian **(**Fig. 1E), or sinusoidal pulse-shapes can be used for neural stimulation^13–15^. The studies indicated that some pulse-shapes may be more energy efficient than rectangular pulses, but that these effects are dependent on pulse width and electrode geometry^16,17^.

The aim of this study was to evaluate and compare the effect of pulse-shape when stimulating in two regions of the rat brain: grey matter in the motor cortex (Experiment 1) and white matter tracts of the deep mesencephalic reticular nucleus (mRT) (Experiment 2). We first designed pulses that were matched in terms of amplitude, pulse width and charge per phase, but differed in shape (Fig. 1). This allows us to evaluate each pulse shape in terms of its charge efficiency. Experiment 1 was conducted in anesthetized rats and used motor cortex stimulation to evoke limb movements allowing us to quickly evaluate a wide range of pulse-shapes. The pulse-shape that produced the largest limb displacement was selected for further testing in Experiment 2 where the outcome measure was immobility levels evoked by stimulating the mRT in awake rats.

## Methods

To evaluate the effect of pulse-shape, two experiments were performed: Experiment 1 focused on motor cortex stimulation in anesthetized rats and used limb accelerometry as an outcome measure, whereas Experiment 2 focused on mRT stimulation in awake rats and used behavioral outcome measures. Both protocols were approved by the KU Leuven ethics committee for laboratory experimentation (project P218/2014), and were in accordance with the Belgian and European laws, guidelines and policies for animal experimentation, housing and care.

### Experiment 1: Motor cortex stimulation

#### Animals

Six male Sprague-Dawley rats (±250 g) were used in Experiment 1. Rats were housed in pairs with food and water available *ad libitum*. Animals were maintained on a 14–10 h light–dark cycle (lights on at 7:00), with a room temperature of approximately 19 °C.

#### Surgery

Rats were anesthetized with a mixture of ketamine (100 mg/kg intraperitoneal, Anesketin, Eurovet, Belgium) and medetomidine HCL (0.2 mg/kg intraperitoneal, Narcostart, Kela Veterinaria, Belgium). Anesthesia was maintained with a perfusion of ketamine (40 mg/kg intraperitoneal). The infusion speed was adjusted as indicated by foot reflex. Microstimulation was performed in the right hemisphere. The primary motor cortex (M1) was exposed by performing a craniotomy going from 5 mm anterior to 5 mm posterior, and 0.5 to 4.5 mm lateral from bregma. After removal of the *dura mater*, a tungsten microelectrode (microTargeting™ electrode, FHC, Inc., USA; 250 µm diameter, impedance between 2 and 10kΩ) was inserted in the caudal forelimb area (AP: 1.5 mm, ML: 2.5 mm, DV: 1.8 mm). This coordinate was determined based on results from an intracortical microstimulation mapping experiment *(unpublished data)*. If stimulation at this location caused no or insufficient forelimb movement, position was adjusted until a satisfactory movement was observed.

#### Electrical Stimulation

Stimulation was delivered using a DS5 current source (Digitimer, Hertfordshire, UK) controlled by a voltage input. The voltage waveform was generated on a data acquisition card (NI USB-6343, National Instruments, Austin, TX) controlled via custom written MATLAB® software (Mathworks, Natwick, MA) at a sampling rate of 200 kHz. This setup allowed the delivery of rectangular, triangular and Gaussian pulse-shapes. Stimulation was delivered between the microelectrode and an anal probe as the return electrode with the first cathodic phase going to the motor cortex.

#### Quantification of limb movement

**Figure 2:**
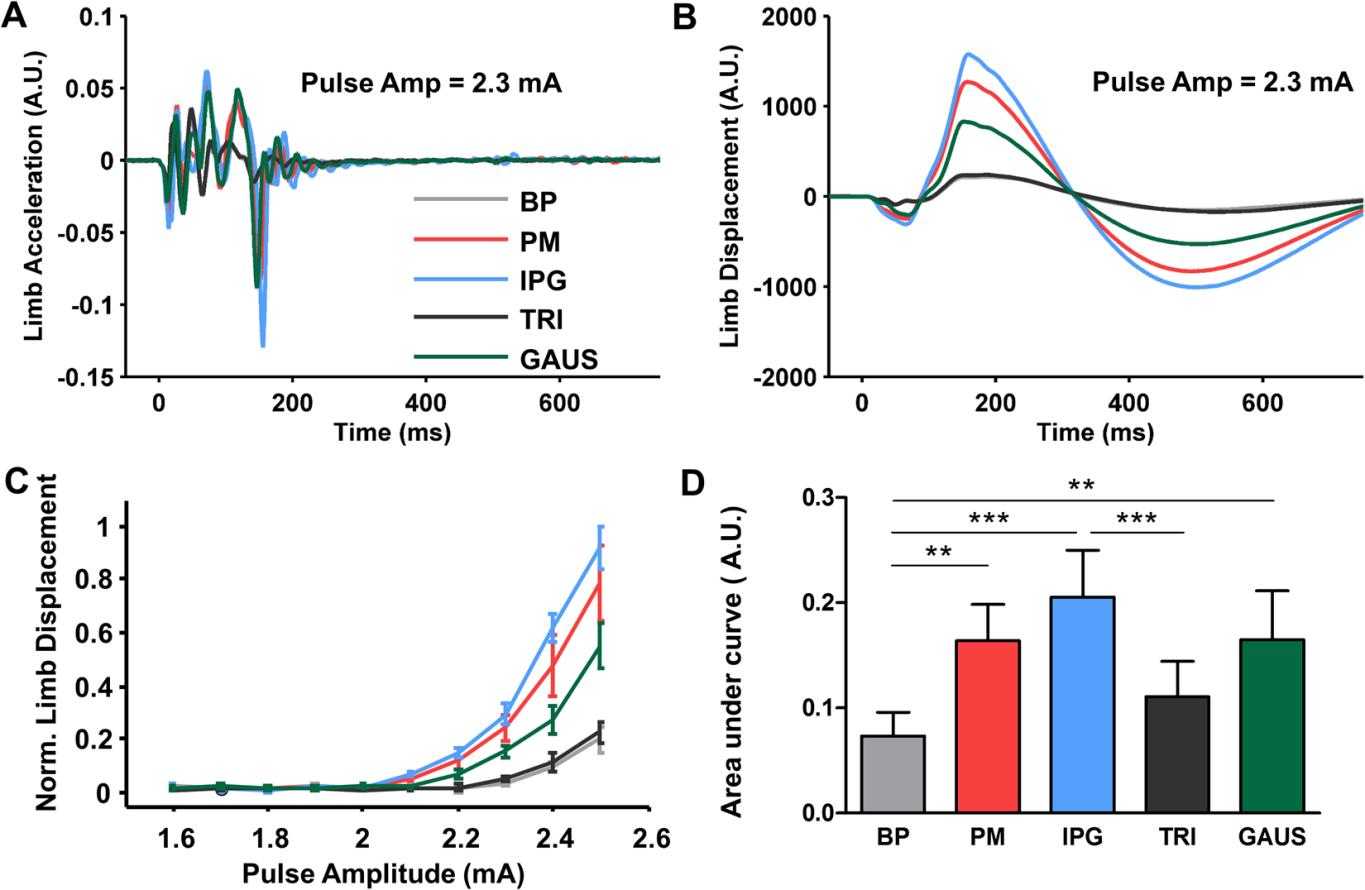
Motor cortex stimulation with different pulse-shapes (Exp. 1, Phase I). (**A**) Shows the bandpass filtered, averaged (5 repetitions), limb acceleration data for the five different pulse-shapes tested at one amplitude. (**B**) Shows the limb displacement data calculated from the acceleration data in panel A. (**C**) Shows the effect of pulse-shape on normalized limb displacement, calculated from the peak in panel B, for a range of pulse amplitudes in one animal. In this rat, biphasic (BP) and triangular (TRI) pulse-shapes produce relatively small limb displacements while Gaussian (GAUS), pseudomonophasic (PM) and interphase gap (IPG) pulse-shapes produce progressively larger displacements for an equivalent pulse amplitude. (**D**) These findings are reflected in the data at the group level (n = 6). Data are shown as means ± SD. **p < 0.01, ***p < 0.001.

To quantify movement, a triaxial accelerometer (ADXL353, Analogue Devices, Norwood, MA) was attached to the forelimb contralateral to the stimulation site. The accelerometer was positioned to have one axis aligned with the principal direction of motion of the limb. The signal was digitized (NI USB-6216, National Instruments, Austin, TX) at 5 kHz and recorded for offline analysis with custom written MATLAB® software (Mathworks, Natwick, MA). The raw acceleration data were bandpass filtered between 1 and 500 Hz (2^nd^ order Butterworth, Fig. 2A) and integrated twice to render the limb displacement in arbitrary units **(**Fig. 2B). The peak limb displacement occurring after stimulation was calculated for each of the three accelerometer axes. The axis showing the largest displacement (i.e. the axis that was aligned with the main direction of motion) was used in all further analysis. Within one animal, the same accelerometer axis was always used to compare all pulse-shapes.

#### Experimental Design

In **Phase I**, five different pulse-shapes were compared – biphasic (BP), pseudomonophasic (PM), interphase gap (IPG), triangular (TRI) and Gaussian (GAUS) **(**Fig. 1**)**. All pulses were charge balanced, *i.e*. for BP, IPG, TRI and GAUS, the anodic and cathodic phases had equal duration and equal but opposite amplitude. Charge per phase was calculated as pulse width times pulse amplitude. Thus, for PM, charge balance was achieved by reducing the amplitude of the anodic phase 10 times, while increasing its duration 10 times. Additionally, all pulse-shapes were designed so that if the amplitude of the cathodic phase was equal, they would also have an equal amount of charge per phase. For TRI and GAUS this meant that the full-width half maximum duration was set equal to the duration of BP but, because of the broader pulse-shape, the interphase gap (as measured from peak to dip in this case) was set equal to the IPG **(**Fig. 1**)**.The cathodic pulse width was set at 100 µs for each pulse-shape. For IPG pulses, the interphase gap was also set at 100 µs. This approach allowed for an investigation of the effect of pulse-shape on limb displacement while keeping pulse width and charge per phase equal between all pulses.

A 300 Hz, 100 ms duration pulse-train was used to elicit limb movement and compare the effect of each pulse-shape. Frequency and pulse width were chosen based on a previous study investigating microstimulation of the motor cortex to induce limb movement^18^ and expertise within our research group *(unpublished data)*. The amplitude of the pulse-train was set to a level below that needed to elicit limb movement (<0.5 mA). The amplitude was then gradually increased by 0.1 mA every 2 seconds, until a robust limb movement was seen (generally between 1 and 2 mA). We obtained a limb displacement response curve with 10 to 12 data points at different pulse amplitudes. This was repeated five times for each pulse-shape, with the repetitions presented in random order. Since limb displacement magnitudes varied between animals, all displacement measurements were normalized to the maximum single limb displacement measured for that animal across all pulse-shapes tested. The normalized data were averaged and plotted as mean ± standard deviation **(**Fig. 2C**)**. Because of the normalization, the displayed limb displacement data are dimensionless.

In **Phase II**, the same procedure was used to test the effect of increasing the interphase gap of a standard rectangular pulse. Five pulse-shapes were tested – biphasic (BP), 50 µs interphase gap (IPG50), 100 µs interphase gap (IPG100), 200 µs interphase gap (IPG200) and 400 µs interphase gap (IPG400). Five of the 6 rats were tested in Phase II.

In **Phase III**, we evaluated pseudomonophasic pulses with various interphase gaps. Five pulse-shapes were tested – standard pseudomonophasic (PM) and pseudomonophasic stimulation with interphase gaps of 50, 100, 200 or 400 µs (PM-IPG50, PM-IPG100, PM-IPG200 and PM-IPG400, respectively). Five of the 6 rats were tested in Phase III.

#### Statistical analysis

To evaluate the effect of stimulation, the area under the curve of the normalized limb displacement response function was calculated for each pulse-shape. For Phase I, a one-way repeated measures analysis of variance (rm-ANOVA) was used to determine if the area under the curve was different for BP, PM, IPG, TRI and GAUS (Bonferroni’s *post hoc* test). This was repeated for Phase II to compare BP, IPG50, IPG100, IGP200 and IPG400 pulses; and for Phase III to compare PM, PM-IPG50, PM-IPG100, PM-IGP200 and PM-IPG400. GraphPad Prism (GraphPad Software, Inc., USA) was used for all analyses and significance was set at p < 0.05.

### Experiment 2: Mesencephalic reticular formation stimulation

#### Animals

Six male Wistar rats (±250 g at time of surgery) were used and housed as in Experiment 1.

#### Surgery

Monopolar electrodes (E363/8, PlasticsOne, Roanoke, VA, USA) were implanted under general anesthesia [ketamine hydrochloride (22.5 mg/kg, Anesketin, Eurovet nv/sa, Heusden-Zolder, Belgium) and 0.15 mg/kg medetomine (Kela, Sint-Niklaas, Belgium)]. Rats were placed in a stereotaxic frame and a craniotomy was performed. Two burr holes were drilled (AP: −5.76 mm; ML: ±1.8 mm) to allow for bilateral electrode insertion in the mRT in the sagittal plane, 5.9 mm subdurally (DV). Four stainless steel screws (Fine Science Tools, Heidelberg, Germany) were inserted in the skull through smaller burr holes. Dental cement (Tetric® EvoFlow, Ivoclar Vivadent Inc., Mississauga, Ontario, Canada) was used to cover the electrodes and the fixation screws before suturing the wound. Animals were allowed to recover for 7 days before the behavioral experiments.

#### Electrical stimulation

Bilateral stimulation was applied in the mRT region, using monopolar electrodes (125 µm diameter Pt/Ir rods (contact size), E363/8, PlasticsOne). A reference screw on the skull served as return electrode. We used a wired, current-controlled stimulation system (STG 4008, Multi Channel Systems MCS GmbH, Reutlingen, Germany) and examined biphasic pulse-shapes with interphase gaps of 0 µs (standard biphasic pulse, BP), 60 µs (IPG60) and 120 µs (IPG120). Frequency and pulse width were set at 130 Hz and 60 µs, respectively, based on a previous study performed by our research group^19^. Stimulation amplitudes were gradually increased in 25 µA steps, to avoid acute behavioral side effects.

#### Experimental design

**Figure 3:**
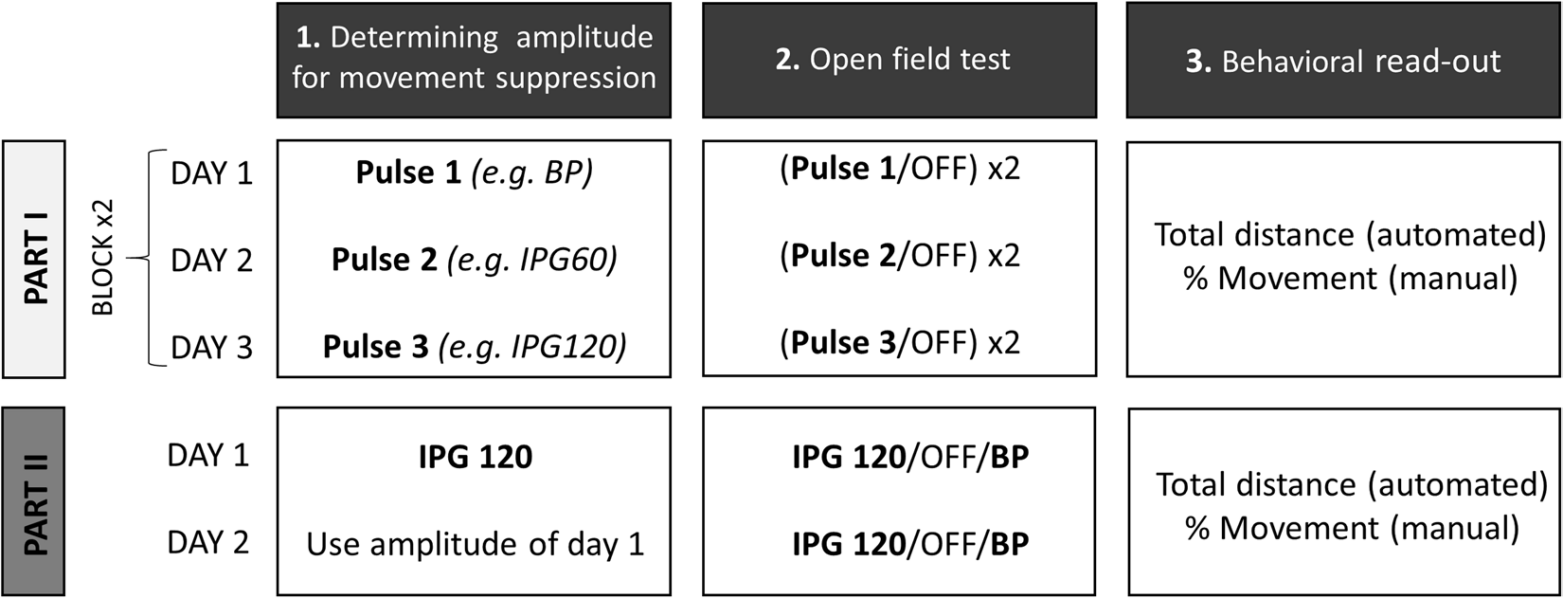
Experimental design for mesencephalic reticular formation stimulation (Experiment 2). In Phase I, we determined the lowest stimulation amplitude that suppressed movement in the home cage, using three different pulses (BP, IPG60, IPG120). The rats were then transferred to the open field, where they received stimulation using this particular pulse shape and amplitude, alternated by OFF conditions. On each test day, only one pulse was evaluated. In total, there were 6 test days for each rat during which each pulse-shape was tested twice. In Phase II, the lowest stimulation amplitude that visibly suppressed mobility was chosen for the IPG120 pulse only. Rats received both BP and IPG120 stimulation in the open field test, using this fixed amplitude. The open field test was repeated the next day, using the same amplitude. Effects on motor behavior were assessed through automated measurements of “Total distance travelled” and manual scores of “%Movement”. Abbreviations: BP = biphasic pulse; IPG60 = interphase gap of 60 µs; IPG120 = interphase gap of 120 µs.

In **Phase I**, we determined the lowest stimulation amplitude that visibly reduced mobility, using three different pulses (BP, IPG60, IPG120). Stimulation amplitudes were gradually increased in 25 µA steps, until immobility was reached in the home cage. The rats were then transferred to the open field, where they received stimulation at this amplitude, alternated by OFF conditions (ON/OFF/ON/OFF; 2 minutes per condition; 1 minute inter-trial interval (ITI)). In total, there were 6 test days for each rat during which each pulse-shape was tested twice **(**Fig. 3**)**. Per test day, only one pulse-shape was evaluated, and pulse-shapes and ON/OFF sequences were randomized. Note that M.D. visually determined immobility thresholds in Phase I and II, while being blinded to the stimulation settings, which were manipulated by K.L. We hypothesized that BP pulses require larger stimulation amplitudes in order to suppress movement to the same extent as the IPG pulses.

In **Phase II**, fixed stimulation parameters were used for BP and IPG120 pulses to assess the behavioral outcome, *i.e*. suppression of movement. For each rat, the lowest stimulation amplitude that visibly suppressed mobility was chosen using the IPG120 pulse. After determination of the amplitude, rats were transferred to the open field and received both BP and IPG120 stimulation using this fixed amplitude (BP/OFF/IPG120; 2 minutes per condition; 1 minute ITI) **(**Fig. 3**)**. We hypothesized that the IPG120 pulse is more effective than BP stimulation, therefore leading to stronger suppression of mobility when keeping the stimulation parameters constant.

#### Video analysis

Movement was quantified by measurements of “Total distance travelled” and “%Movement”. “Total distance travelled” was automatically detected through an in-house developed algorithm^19,20^. Movement was scored manually as the percentage in time, with respect to the total duration of the session, during which the rat moved at least one limb (*e.g*. during locomotion and grooming) or displayed the stretch-attend posture.

#### Histology

One week after testing, rats were given a lethal intraperitoneal injection of pentobarbital (2 ml, Nembutal, CEVA Santé Animale, Belgium). The animals were perfused with a solution of 10% sucrose (D(+)-Saccharose, VWR BVBA, Leuven, Belgium), and subsequently with a 4% formaldehyde solution (37% dissolved in water, stabilized with 5–15% methanol, Acros organics, Belgium, 10× diluted in DI water). The brains were dissected and stored in 4% formaldehyde, processed in the Histostar and embedded in paraffin. Slices (5 µm) were collected with the microtome and stained with Cresyl-Violet (0.5% cresyl violet acetate in dH2O, Merck KGaA, Germany). Microscopic analysis revealed the location of electrode tips and lesions, which were transferred to a 2D Paxinos plate.

#### Statistical analysis

One-way repeated measures analysis of variance (rm-ANOVA) was used to examine whether immobility-inducing stimulation amplitudes differed between pulse-shapes (Phase I) and to evaluate the effect of pulse-shape on motor outcome (Phase II). Bonferroni’s’s *post hoc* tests were used to identify group differences. Two-way rm-ANOVA was used to examine whether electrical stimulation had an effect on the motor measurements in Phase I (stimulation ON vs OFF for BP, IPG60 and IPG120, Bonferroni post hoc test). A two-sided Grubbs test was used to identify any outliers. GraphPad Prism (GraphPad Software, Inc., USA) was used for all analyses and significance was set at p < 0.05.

## Results

### Experiment 1: Motor cortex stimulation

In **Phase I**, we used rat motor cortex to evaluate the effect of pulse-shape (BP, PM, IPG, GAUS and TRI) on limb displacement. In line with similar studies using standard BP pulses^21^, the average stimulation amplitude needed for a BP pulse to elicit a selective limb movement was 1.5 mA. Figure 2C shows the limb displacement response curve as a function of pulse amplitude for one representative rat. For each pulse-shape, limb displacement increases monotonically with increasing pulse amplitude. At a given pulse amplitude, the limb displacement response varies depending on the pulse-shape. In this rat, for a fixed pulse amplitude, BP and TRI produced the smallest displacements, while GAUS, PM and IPG produced progressively larger displacements. For each rat, the area under the curve was calculated for all pulse-shapes and group data from six rats are shown in Fig. 2D. With the exception of TRI, all pulse-shapes had a significantly larger area under the curve than the standard BP (main effect of pulse-shape: F(4, 6) = 14.58, p < 0.0001; Bonferroni’s *post hoc* test: p < 0.01 for BP vs PM; p < 0.001 for BP vs IPG; p > 0.05 for BP vs TRI; p < 0.01 for BP vs GAUS and p < 0.001 for IPG vs TRI). This indicates that PM, IPG and GAUS pulse-shapes produce a significantly larger displacement than a BP pulse-shape of equal amplitude. For any given pulse amplitude, all pulse-shapes were designed to have the same amount of charge per phase. Therefore, we can also conclude that PM, IPG and GAUS are more effective than a BP pulse with an equal amount of charge.

**Figure 4:**
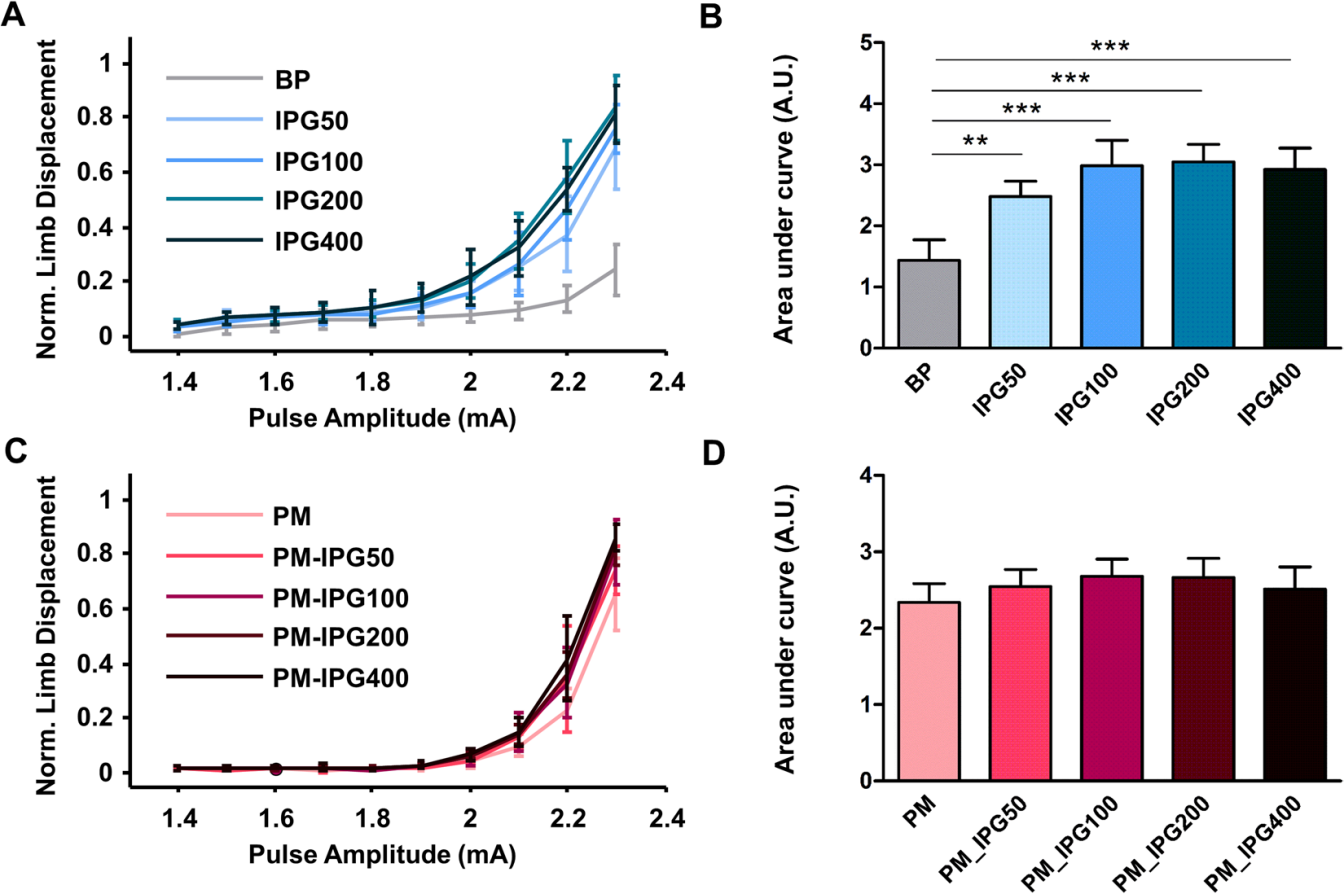
Motor cortex stimulation with different interphase gaps (Exp. 1, Phase II (**A**,**B**) and Phase III (**C**,**D**)). (**A**) Shows the effect of interphase gap duration within a biphasic pulse on normalized limb displacement for one subject. In this rat, introducing a 50 µs interphase gap (IPG50) produced a larger limb displacement when compared to a biphasic pulse with no interphase gap (BP). Increasing the interphase gap to 100 µs (IPG100) only led to a small increase in limb displacement. Further increase in interphase gap to 200 and 400 µs (IPG200, IPG400) did not lead to further increases in limb displacement. (**B**) These findings are reflected in the data at the group level (n = 5). (**C**) Shows the effect of interphase gap duration within a pseudomonophasic pulse on normalized limb displacement for one individual rat. In this rat, introducing a 50 µs interphase gap (PM_IPG50) produced only a very small increase in limb displacement when compared to a pseudomonophasic pulse with no interphase gap (PM). Further increases in interphase gap to 100, 200 and 400 µs (PM-IPG100, PM-IPG200, PM-IPG400) did not lead to further increases in limb displacement. (**D**) These findings are reflected in the data at the group level (n = 5). Data are shown as means ± SD. **p < 0.01, ***p < 0.001. Abbreviations: BP = biphasic, IPG = interphase gap, PM = pseudomonophasic.

**Phase II** investigated the effect of the interphase gap duration. Data from one representative rat are shown in Fig. 4A. Here, IPG50 produced a significant increase in limb displacement compared to BP. IPG100 and IPG200 produced small additional increases in displacement. However, displacement did not increase further for IPG400. At the group level (Fig. 4B) IPG50, IPG100, IPG200 and IPG400 all showed a significant increase compared to BP, but did not differ significantly from each other (main effect of IPG: F(4, 5) = 19.08, p < 0.0001; Bonferroni’s *post hoc* test: p < 0.01 for BP vs IPG50; p < 0.001 for BP vs IPG100; p < 0.001 for BP vs IPG200; p < 0.001 for BP vs IPG400).

**Phase III** examined the effect of adding an interphase gap in the PM pulse. The results from one rat are shown in Fig. 4C and the group results in Fig. 4D. Inserting an interphase gap into the PM pulse produced only small increases in limb displacement compared to a PM without IPG. Adding an interphase gap to PM did not produce a significant increase in displacement (F(4, 5) = 1.7, p = 0.2).

### Experiment 2: Mesencephalic reticular formation stimulation

The behavioral testing methods used in Experiment 2 require considerably more experimental resources than the accelerometry method used in Experiment 1. Therefore, only the most effective pulse-shape (IPG) was selected for testing. Two IPGs (60 µs and 120 µs) were chosen to cover a range where we expected the most interesting results.

#### Histology

**Figure 5:**
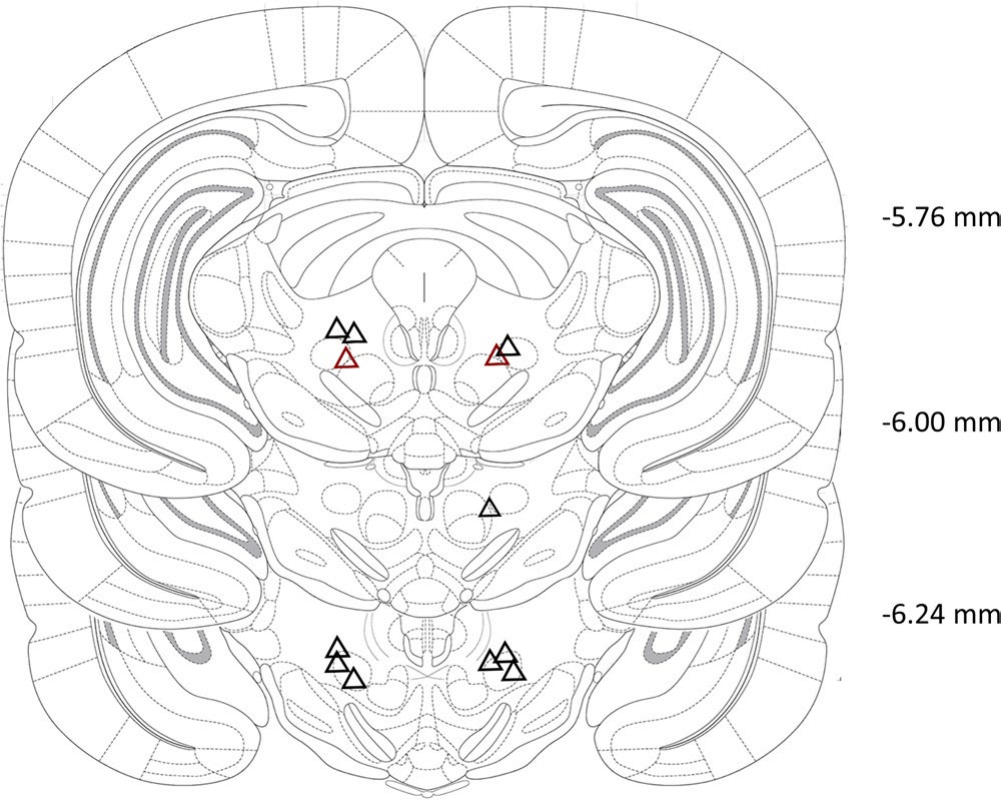
Schematic representation of the electrode tip localization (triangles) in the mRT region (Exp. 2). Anterior-posterior coordinates are shown with respect to bregma. Red triangles represent electrode location of one animal which was omitted from behavioral analysis (see discussion). Adapted with permission from Paxinos, G. & Watson, C. “The rat brain in stereotaxic coordinates.” (Bregma −5.76 mm, Bregma −6.00 mm and Bregma −6.24 mm; Elsevier Academic Press, 2005).

All electrodes were implanted in the intended target **(**Fig. 5**)**.

#### Behavioral results

The Grubb’s test revealed one rat as a significant outlier for both “Total distance travelled” and “% movement” in Phase I and in Phase II. Therefore, this rat was excluded from all analyses (Supplementary Figure 1).

**Figure 6:**
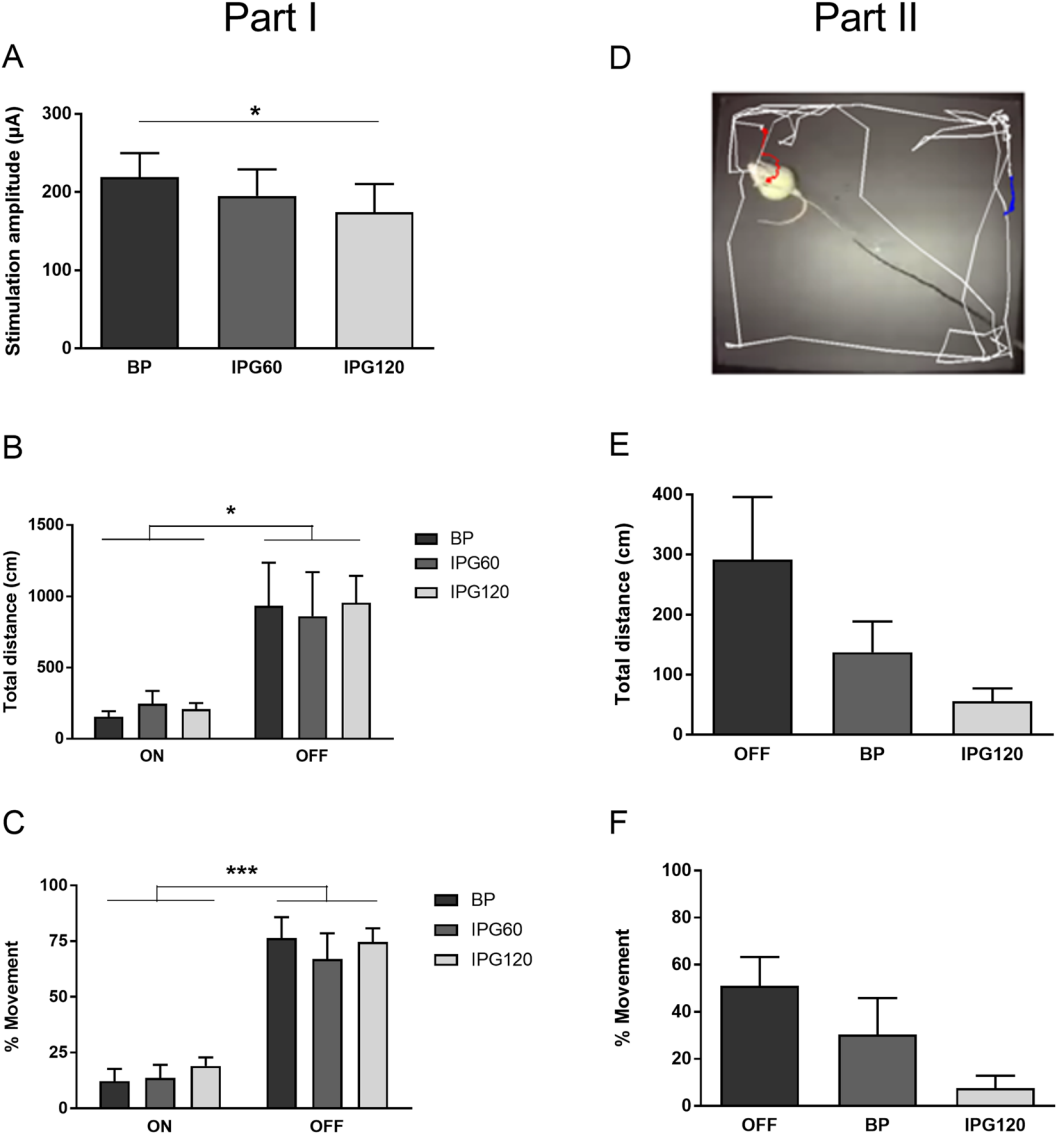
mRT stimulation (Experiment 2, phase I (**A**–**C**) and phase II (**D**–**F**). In Phase I, we found that introducing an IPG of 120 µs significantly decreased the minimal amplitude required to visibly suppress movement, compared to BP stimulation (**A**). All three pulses were equally successful in reducing both “Total distance” (**B**) and “%Movement” (**C**) at their individually set threshold amplitude. (**D**) Shows automated tracking of the total distance travelled by a representative rat in BP (red), IPG120 (blue) and OFF (white) conditions. In phase II, BP and IPG120 stimulation at fixed amplitudes did not generate significantly differential effects on motor outcome, although visual inspection of the data suggests that IPG120 stimulation is the most efficacious (**E**,**F**). Data are shown as mean ± SEM (n = 5 for each group, crossover design). *p < 0.05, ***p < 0.001. Abbreviations: BP = biphasic pulse; IPG60 = interphase gap of 60 µs; IPG120 = interphase gap of 120 µs.

In **Phase I**, we determined the lowest amplitude necessary to suppress movement for three pulse-shapes: BP, IPG60 and IPG120. The average BP amplitude needed to observe a behavioral effect in the mRT was in line with values reported in studies using electrical stimulation in the ventral striatum^22^ and limbic system^23^. We found that significantly lower stimulation amplitudes were necessary when introducing an interphase gap of 120µs (IPG120 vs BP, (F(2, 4) = 7.30, p = 0.02)) **(**Fig. 6A**)**. An interphase gap of 60 µs required lower stimulation amplitudes compared to BP but the difference was not significant. Note that all stimulation pulses at their individually determined amplitudes suppressed the total distance travelled and percentage movement to the same extent (main effect of stimulation F(1, 4) = 7.66, p = 0.02 and F(1, 4) = 40.06, p < 0.001 respectively; Fig. 6B and C).

In **Phase II**, stimulation efficacy was evaluated in terms of movement suppression, using fixed amplitudes for both BP and IPG120 pulses. Figure 6D shows automated tracking of the total distance travelled by a representative rat in BP (red), IPG120 (blue) and OFF (white) conditions. Here, we found that neither BP nor IPG120 stimulation had a significant effect on “Total distance travelled” (F(2, 4) = 2.69, p = 0.13, Fig. 6E) and “% Movement” (F(2, 4) = 3.57, p = 0.08, Fig. 6F). However, this lack of significance can be attributed to increased variability in behavioral data, compared with Phase I (see ‘Discussion’).

## Discussion

This is the first study that uses *in vivo* stimulation in both grey and white matter to examine pulse-shape effects; evaluating both rectangular and non-rectangular pulses. Below we discuss the results obtained for each of the pulse-shapes.

### Interphase gap

Interphase gap is one of the best studied pulse-shapes in auditory nerve stimulation. Some cochlear implant systems already use an interphase gap to increase stimulation effectiveness and prolong battery life^24^. Some commercial DBS systems also employ an IPG in combination with a passive recharge phase^25^. In spite of this clinical use, studies looking at the effect of IPG using *in vivo* grey or white matter stimulation are few. One study using suprachoroidal electrical stimulation in cats where stimulation parameters for use in a retinal prosthesis were explored, found that an interphase gap could reduce the threshold for activation of the visual cortex^26^. These results were confirmed by a modeling study on retinal stimulation^27^.  In addition, an *in vitro* study by Cappaert *et al*.^28^ showed that IPG pulses were superior during stimulation of the rat sciatic nerve and hippocampal slices for both constant voltage and current stimulation.

In Experiment 1 we showed that when delivering equal amounts of charge per phase to the motor cortex, IPG caused the largest limb displacement. Experiment 2 evaluated the effect of IPG on white matter mRT stimulation and found a similar effect in Phase I: IPG120 required significantly less charge to suppress movement than BP. Phase II use a fixed pulse-amplitude to examine this effect. The data suggested that both BP and IPG120 reduced movement compared with the OFF condition, with IPG120 being most effective. However, the results failed to reach significance, probably because of increased measurement variability. Due to habituation to the testing environment, some rats were no longer exploring the open field during the OFF condition, making it difficult to detect a suppression of movement and leading to increased variability. Additionally, measurements in the BP condition were confounded by ‘circling’ behavior, a known effect of mRT stimulation^29^ which we observed at amplitudes that were not sufficient to suppress movement. Finally, we note that one animal was excluded from all analyses in both Part I and Part II, since it displayed aberrant behavior during stimulation intervals (Supplementary Figure 1). We were unable to reach full immobility in this animal, but instead observed that the animal appeared absent (i.e. did not respond to any auditory or visual trigger) and attended an upwards position upon stimulation. This behavior was observed at both low and high stimulation amplitudes. Although it is possible that immobility could have been reached at higher amplitudes, this might have endangered the animal’s well-being (e.g. stimulation-induced seizure) and was therefore not explored.

Note that the stimulation targets used in Experiments 1 and 2 clearly differed – one was a grey matter cortical area, the other a white matter deep brain region. Additionally, we used different animal preparations, rat strains and outcome measures. In spite of these dissimilar procedures, the results from both experiments agree – introducing an interphase gap in a biphasic pulse produces more effective stimulation. Taken together with the previously published studies reviewed above, our results indicate that the use of an interphase gap is a general approach for increasing the effect of stimulation that can be applied across a range of different neural populations, including the central nervous system.

#### Pseudomonophasic

Experimental studies on auditory nerve stimulation have shown that PM pulses require lower amplitudes to achieve the same response as BP pulses^10–12^. Results from Experiment 1, Phase I show that this is also the case when PM pulses are used for grey matter motor cortex stimulation. In Phase III, we investigated if combining an IPG with a PM pulse could produce further improvements but did not find a significant effect. This is seemingly in contradiction with a modeling study by Hofmann *et al*.^30^, who tested the effect of introducing an IPG into a PM pulse and found that it should increase stimulation efficiency for deep brain stimulation. If the effect in our experiment was small, it could be obscured by noise in the experimental setup (*i.e*. small movements in electrode or limb position) and therefore remain undetected. It is also important to point out that Hofmann *et al*. observed the largest effects of combining PM with IPGs of 2 ms. In our study we did not test these very long IPGs. In addition, our target region was the motor cortex (i.e. grey matter), while the modeling study of Hofmann *et al*. primarily relied on axonal activation properties.

### Non-rectangular pulses (TRI, GAUS)

The non-rectangular GAUS pulse-shape produced a larger limb displacement than a BP pulse-shape with an equivalent charge per phase. However, for the equivalent charge per phase, one GAUS or TRI phase had a longer duration than one BP or IPG phase, meaning that GAUS and TRI always had an interphase gap (as measured from peak to dip) equivalent to IPG. Therefore, when assessing the results it is fairest to compare GAUS and TRI with IPG. Accordingly IPG was the most effective, giving a significantly larger limb displacement than TRI but not significantly larger than GAUS (Fig. 2D). These findings are of particular interest given the recent work of Ballestero *et al*.^17^, who showed that a pulse-shape with a ramped top could improve the spatial selectivity when stimulating spiral ganglion neurons *in vitro*. It is also important to point out that in this set of experiments we did not investigate the effect of different interphase gap durations with the non-rectangular pulse shapes. Optimizing this parameter for non-rectangular pulse shapes may lead to further improvements in charge efficiency.

### Potential for application in patients

All commercially available neural implants currently use rectangular pulse-shapes for stimulation. Most commercial DBS devices typically use a passive recharge phase resulting in a pulse-shape somewhere between the BP and PM pulses used in this study^25^. Interestingly, a recent study highlighted some potential advantages of using a standard BP pulse, with an active recharge phase, in DBS patients^31^. Our study showed that using IPG and PM pulses can significantly increase responses when compared to BP pulses. A number of commercially available DBS devices already have the capability to deliver IPG and PM pulses, meaning that these pulse-shapes could be used in patients without requiring any changes to the current device hardware.

From a hardware perspective, it is simpler to design a neural implant that delivers rectangular, as opposed to non-rectangular, pulses shapes. Thus, the use of rectangular pulse-shapes is mainly driven by limitations in electrical circuitry of the devices and not experimental evidence suggesting their superiority. The data presented in our study show that it is possible to use non-rectangular pulse-shapes for brain stimulation but more work is needed to investigate their clinical relevance. Our study focused on evaluating the effect of pulse-shape for grey and white matter stimulation. However, we should also note that factors such as electrode geometry, pulse width, frequency and constant current versus constant voltage stimulation can influence the effectiveness of a particular pulse-shape^13,28^. In this study we selected stimulation frequencies for the motor cortex and mRT based on values from the literature^18,19^ and kept these fixed while we changed pulse shape. More studies are needed to confirm that the effects of pulse shape observed at these frequencies also hold for other stimulation frequencies.

## Conclusion

The results from this study help establish what appears to be a general principle of electrical stimulation of the nervous system – *i.e*. introducing an interphase gap into a biphasic pulse can produce a larger response without having to increase the amount of charge. In addition, we showed that non-rectangular pulse-shapes can be used for *in vivo* brain stimulation. However, further evaluation is needed to determine their potential use in clinical devices and to explore the effect of all pulse parameters. Additionally, these findings have important implications for modeling studies which seek to understand how a neuron integrates injected charge to fire an action potential.

### Significance Statement

Neural implants benefit hundreds of thousands of people by restoring a sense or treating a neurological disorder. They work by injecting charge into the nervous system, which in most cases depolarizes the membrane and initiates an action potential. Charge is typically injected using a biphasic rectangular pulse. Evidence from peripheral stimulation of auditory nerve shows that reducing the amplitude or delaying the second phase of a biphasic pulse can increase stimulation efficiency. Theoretical studies also indicate that non-rectangular pulses such as triangular or Gaussian pulses may be beneficial in neural implants. In this study we used electrical stimulation of both grey and white targets in the rat brain to show that delaying the second phase of a rectangular pulse is a general principle for improving neural stimulation charge efficiency. In the motor cortex, we showed that Gaussian pulses were also an effective stimulation mode. Our results have important implications for the understanding of electrical stimulation of the nervous system and open new perspectives for the design of the next generation of safe and efficient neural implants.
